# (μ-Di­hydrogen pyrazine-2,3,5,6-tetra­carboxyl­ato-κ^6^ *O* ^2^,*N* ^1^,*O* ^6^;*O* ^3^,*N* ^4^,*O* ^5^)bis­(di­aqua­lithium) monohydrate

**DOI:** 10.1107/S1600536814007223

**Published:** 2014-04-09

**Authors:** Wojciech Starosta, Janusz Leciejewicz

**Affiliations:** a Institute of Nuclear Chemistry and Technology, ul.Dorodna 16, 03-195 Warszawa, Poland

## Abstract

The structure of the title compound, [Li_2_(C_8_H_2_N_2_O_8_)(H_2_O)_4_]·H_2_O, is composed of dinuclear mol­ecules in which the ligand bridges two symmetry-related Li^I^ ions, each coordinated also by two water O atoms, in an *O*,*N*,*O*′-manner. The Li and N atoms occupy special positions on twofold rotation axes, whereas a crystal water mol­ecule is located at the inter­section of three twofold rotation axes. The Li^I^ cation shows a distorted trigonal–bipyramidal coordination. Two carboxyl­ate groups remain protonated and form short inter­ligand hydrogen bonds. The mol­ecules are held together by a network of hydrogen bonds in which the coordinating and solvation water mol­ecules act as donors and carboxyl­ate O atoms as acceptors, forming a three-dimensional architecture.

## Related literature   

For the structure of a lithium complex with pyrazine-2,3,5,6-tetra­carboxyl­ate and water ligands, see: Starosta & Leciejewicz (2010 ▶). The structure of pyrazine-2,3,5,6-tetra­carb­oxy­lic acid dihydrate was reported by Vishweshwar *et al.* (2001 ▶).
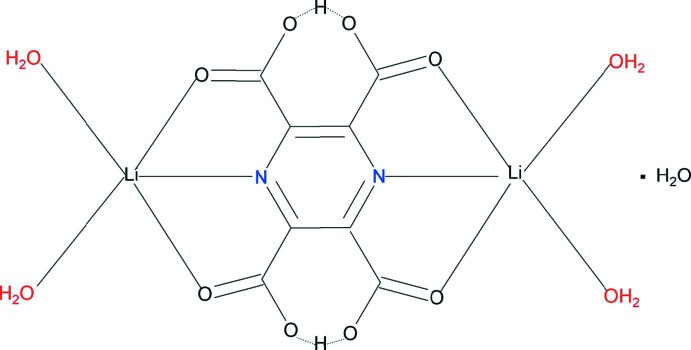


## Experimental   

### 

#### Crystal data  

[Li_2_(C_8_H_2_N_2_O_8_)(H_2_O)_4_]·H_2_O
*M* *_r_* = 358.08
Orthorhombic, 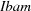

*a* = 6.3807 (4) Å
*b* = 9.8331 (6) Å
*c* = 22.1717 (16) Å
*V* = 1391.10 (16) Å^3^
*Z* = 4
Mo *K*α radiation
μ = 0.16 mm^−1^
*T* = 293 K
0.12 × 0.10 × 0.06 mm

#### Data collection  

Agilent SuperNova (Dual, Cu at zero, Eos) diffractometer
Absorption correction: multi-scan (*CrysAlis PRO*; Agilent, 2011 ▶) *T*_min_ = 0.979, *T*_max_ = 0.993
3622 measured reflections
917 independent reflections
769 reflections with *I* > 2σ(*I*)
*R*_int_ = 0.061

#### Refinement  

*R*[*F* ^2^ > 2σ(*F* ^2^)] = 0.047
*wR*(*F* ^2^) = 0.100
*S* = 1.06
917 reflections
78 parameters
4 restraints
H atoms treated by a mixture of independent and constrained refinement
Δρ_max_ = 0.25 e Å^−3^
Δρ_min_ = −0.22 e Å^−3^

### 

Data collection: *CrysAlis PRO* (Agilent, 2011 ▶); cell refinement: *CrysAlis PRO*; data reduction: *CrysAlis PRO*; program(s) used to solve structure: *SHELXS97* (Sheldrick, 2008 ▶); program(s) used to refine structure: *SHELXL97* (Sheldrick, 2008 ▶); molecular graphics: *SHELXTL* (Sheldrick, 2008 ▶); software used to prepare material for publication: *SHELXTL*.

## Supplementary Material

Crystal structure: contains datablock(s) I, New_Global_Publ_Block. DOI: 10.1107/S1600536814007223/kp2468sup1.cif

Structure factors: contains datablock(s) I. DOI: 10.1107/S1600536814007223/kp2468Isup2.hkl

CCDC reference: 994866

Additional supporting information:  crystallographic information; 3D view; checkCIF report

## Figures and Tables

**Table 1:** Selected bond lengths (Å)

Li1—N1	2.053 (4)
Li1—O1	2.1581 (17)
Li1—O3	1.969 (3)
Li1—O3^i^	1.969 (3)
Li1—O1^i^	2.1580 (17)

Symmetry code: (i) 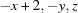.

**Table 2:** Hydrogen-bond geometry (Å, °)

*D*—H⋯*A*	*D*—H	H⋯*A*	*D*⋯*A*	*D*—H⋯*A*
O2—H1⋯O2^ii^	1.20 (1)	1.20 (1)	2.402 (3)	177 (8)
O3—H32⋯O3^iii^	0.87 (2)	2.00 (2)	2.839 (3)	163 (4)
O3—H31⋯O1^iv^	0.86 (2)	2.03 (2)	2.8825 (19)	177 (2)
O3—H33⋯O4^v^	0.86 (2)	2.10 (2)	2.9454 (17)	169 (6)

Symmetry codes: (ii) 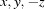; (iii) 
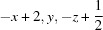
; (iv) 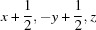; (v) 
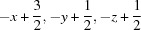
.

**Fig. 1.**
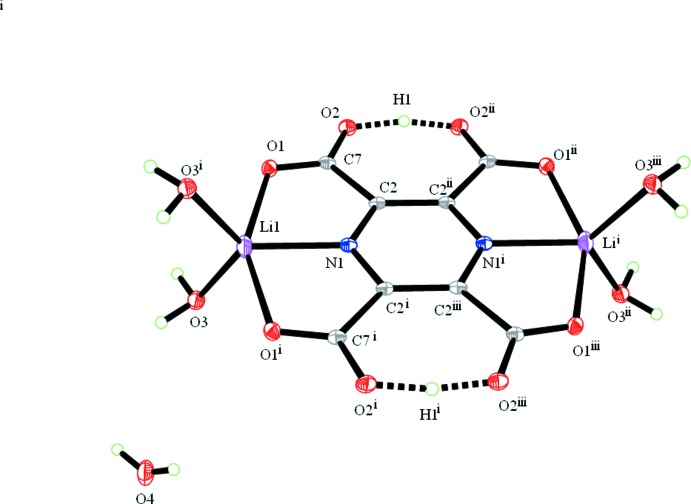
The molecule of the title compound with atom labelling scheme and 50% probability displacement ellipsoids. Symmetry code: i -x, -y, z; iix, y, -z; iii -x, -y, -z.

**Fig. 2.**
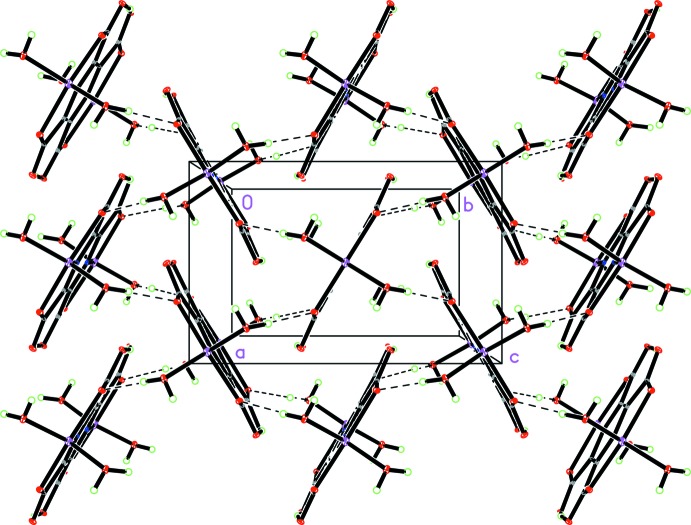
A single molecular layer composed of the dimers linked by a hydrogen bond network as viewed along the crystal c direction.

## supplementary crystallographic information

### 1. Comment

The structure of the title compound reveals a dimeric unit of 2/m crystallographic symmetry in which two symmetry related doubly aqua-coordinated Li(I) ions are bridged by a ligand molecule *via* its both N,*O*,*O* bonding sites (Fig.1). The Li(I) cation is in distorted trigonal bipyramidal coordination. Its equatorial plane is formed by N1, O3, O3^(i)^ and the Li1 atoms; the O1 and O1^(i)^ atoms are at the apices. The O1—Li—O1^(i)^ angle is 151.7 (3)° and Li—O and Li—N bond distances are listed in Table 1. The pyrazine ring is planar. Two carboxylate groups remain protonated to maintain charge balance. Bond distances and bond angles within the hetero-ring do not differ from those reperted in the structure of the parent acid (Vishweshwar *et al.*, 2001). Short, symmetric hydrogen bond is formed in which the carboxylate O2 atom is as a donor and the O1^(ii)^ is as an acceptor (Table 2). A dihedral angle formed with the pyrazine ring by the carboxylic group C1/O1/O2 is 4.7 (1)°. Fourier maps show a clear disorder in hydrogen positions at the solvation and coordinated water molecules with position occupancy of aproximately 0.5. The title molecules are held together by a network of hydrogen bonds (Table 2) in which coordinated water molecules act as donors and carboxylate O atoms as acceptors forming molecular layers propagating in the crystal *a* direction (Fig.2). A view of a single layer is displayed in Fig. 2. The layers are linked by weak hydrogen bonds in which solvation water molecules are involved. Another Li(I) complex with the title ligand is known (Starosta & Leciejewicz, 2010). Its polarized dinuclear structural unit is built of a doubly protonated ligand molecule chelated by its N,*O*,*O* bonding moiety to an Li(I) ion and an Li(I) ion coordinated by four aqua O atoms. Two of the latter bridge the Li ions to form a catenated polymeric structure propagating in the crystal *a* direction.

### 2. Experimental

Hot aqueous solutions, one containing 1 mmol of pyrazine-2,3,5,6-tetracarboxylic acid dihydrate, the other 4 mmol s of lithium nitrate (Aldrich) were mixed and boiled under reflux with constant stirring for 6 h. After cooling to room temperature the solution was left to evaporate. A couple of days latter, single-crystal blocks deposited on the bottom of a crystallization pot. They were washed with cold ethanol and dried in air.

### 3. Refinement

Water and carboxylate H atoms were found in the Fourier map and refined isotropically.

### Crystal data

**Table:**

[Li_2_(C_8_H_2_N_2_O_8_)(H_2_O)_4_]·H_2_O	*Z* = 4
*M**_r_* = 358.08	*F*(000) = 736
Orthorhombic, *I**b**a**m*	*D*_x_ = 1.710 Mg m^−^^3^
Hall symbol: -I 2 2c	Mo *K*α radiation, λ = 0.71073 Å
*a* = 6.3807 (4) Å	µ = 0.16 mm^−^^1^
*b* = 9.8331 (6) Å	*T* = 293 K
*c* = 22.1717 (16) Å	Plate, colourless
*V* = 1391.10 (16) Å^3^	0.12 × 0.10 × 0.06 mm

### Data collection

**Table:**

Agilent SuperNova (Dual, Cu at zero, Eos) diffractometer	917 independent reflections
Radiation source: SuperNova (Mo) X-ray Source	769 reflections with *I* > 2σ(*I*)
Mirror monochromator	*R*_int_ = 0.061
Detector resolution: 16.0131 pixels mm^-1^	θ_max_ = 29.3°, θ_min_ = 3.7°
ω scans	*h* = −8→8
Absorption correction: multi-scan (*CrysAlis PRO*; Agilent, 2011)	*k* = −12→13
*T*_min_ = 0.979, *T*_max_ = 0.993	*l* = −29→29
3622 measured reflections	

### Refinement

**Table:**

Refinement on *F*^2^	Primary atom site location: structure-invariant direct methods
Least-squares matrix: full	Secondary atom site location: difference Fourier map
*R*[*F*^2^ > 2σ(*F*^2^)] = 0.047	Hydrogen site location: inferred from neighbouring sites
*wR*(*F*^2^) = 0.100	H atoms treated by a mixture of independent and constrained refinement
*S* = 1.06	*w* = 1/[σ^2^(*F*_o_^2^) + (0.024*P*)^2^ + 2.020*P*] where *P* = (*F*_o_^2^ + 2*F*_c_^2^)/3
917 reflections	(Δ/σ)_max_ < 0.001
78 parameters	Δρ_max_ = 0.25 e Å^−^^3^
4 restraints	Δρ_min_ = −0.22 e Å^−^^3^

### Special details

**Table:**

Geometry. All e.s.d.'s (except the e.s.d. in the dihedral angle between two l.s. planes) are estimated using the full covariance matrix. The cell e.s.d.'s are taken into account individually in the estimation of e.s.d.'s in distances, angles and torsion angles; correlations between e.s.d.'s in cell parameters are only used when they are defined by crystal symmetry. An approximate (isotropic) treatment of cell e.s.d.'s is used for estimating e.s.d.'s involving l.s. planes.
Refinement. Refinement of *F*^2^ against ALL reflections. The weighted *R*-factor *wR* and goodness of fit *S* are based on *F*^2^, conventional *R*-factors *R* are based on *F*, with *F* set to zero for negative *F*^2^. The threshold expression of *F*^2^ > σ(*F*^2^) is used only for calculating *R*-factors(gt) *etc*. and is not relevant to the choice of reflections for refinement. *R*-factors based on *F*^2^ are statistically about twice as large as those based on *F*, and *R*- factors based on ALL data will be even larger.

### Fractional atomic coordinates and isotropic or equivalent isotropic displacement parameters (Å^2^)

**Table:**

	*x*	*y*	*z*	*U*_iso_*/*U*_eq_	Occ. (<1)
N1	1.0000	0.0000	0.06097 (8)	0.0207 (4)	
O1	0.7218 (2)	0.11263 (14)	0.12977 (5)	0.0328 (3)	
O3	1.1372 (3)	0.14626 (15)	0.19959 (7)	0.0376 (4)	
O2	0.5112 (3)	0.16787 (13)	0.05418 (6)	0.0356 (4)	
C2	0.8398 (3)	0.05696 (15)	0.03197 (7)	0.0203 (3)	
C7	0.6800 (3)	0.11721 (16)	0.07586 (8)	0.0251 (4)	
Li1	1.0000	0.0000	0.15359 (19)	0.0360 (10)	
O4	0.0000	0.5000	0.2500	0.0595 (9)	
H31	1.158 (4)	0.219 (2)	0.1789 (10)	0.056 (8)*	
H1	0.506 (12)	0.168 (4)	0.0000	0.097 (15)*	
H32	1.068 (6)	0.161 (4)	0.2328 (13)	0.037 (13)*	0.50
H41	0.043 (12)	0.459 (6)	0.2190 (17)	0.10 (3)*	0.50
H33	1.254 (6)	0.111 (6)	0.211 (2)	0.08 (2)*	0.50

### Atomic displacement parameters (Å^2^)

**Table:**

	*U*^11^	*U*^22^	*U*^33^	*U*^12^	*U*^13^	*U*^23^
N1	0.0218 (8)	0.0189 (8)	0.0214 (9)	0.0016 (8)	0.000	0.000
O1	0.0355 (7)	0.0383 (7)	0.0244 (6)	0.0065 (6)	0.0072 (6)	−0.0025 (5)
O3	0.0474 (9)	0.0347 (8)	0.0308 (8)	−0.0074 (7)	−0.0008 (7)	0.0004 (6)
O2	0.0293 (7)	0.0400 (7)	0.0376 (7)	0.0154 (6)	0.0050 (7)	0.0002 (6)
C2	0.0198 (8)	0.0174 (7)	0.0239 (8)	0.0002 (6)	0.0008 (6)	−0.0009 (6)
C7	0.0258 (8)	0.0203 (7)	0.0293 (9)	0.0019 (6)	0.0047 (7)	−0.0008 (6)
Li1	0.046 (2)	0.039 (2)	0.024 (2)	−0.005 (2)	0.000	0.000
O4	0.076 (3)	0.065 (2)	0.0372 (19)	0.000	0.000	0.000

### Geometric parameters (Å, º)

**Table:**

N1—C2^i^	1.3313 (18)	O2—C7	1.280 (2)
N1—C2	1.3313 (18)	O2—H1	1.202 (3)
Li1—N1	2.053 (4)	C2—C2^ii^	1.418 (3)
O1—C7	1.226 (2)	C2—C7	1.529 (2)
Li1—O1	2.1581 (17)	Li1—O3^i^	1.969 (3)
Li1—O3	1.969 (3)	Li1—O1^i^	2.1580 (17)
O3—H31	0.858 (16)	Li1—H33	2.33 (5)
O3—H32	0.870 (19)	O4—H41	0.84 (2)
O3—H33	0.86 (2)		
			
C2^i^—N1—C2	122.24 (19)	O2—C7—C2	118.22 (15)
C2^i^—N1—Li1	118.88 (10)	O3^i^—Li1—O3	117.6 (2)
C2—N1—Li1	118.88 (10)	O3^i^—Li1—N1	121.21 (11)
C7—O1—Li1	115.89 (16)	O3—Li1—N1	121.21 (11)
Li1—O3—H31	113.4 (17)	O3^i^—Li1—O1^i^	96.76 (7)
Li1—O3—H32	110 (3)	O3—Li1—O1^i^	97.81 (7)
H31—O3—H32	113 (3)	N1—Li1—O1^i^	75.84 (11)
Li1—O3—H33	104 (4)	O3^i^—Li1—O1	97.81 (7)
H31—O3—H33	111 (4)	O3—Li1—O1	96.76 (7)
H32—O3—H33	105 (4)	N1—Li1—O1	75.84 (11)
C7—O2—H1	113 (4)	O1^i^—Li1—O1	151.7 (2)
N1—C2—C2^ii^	118.88 (10)	O3^i^—Li1—H33	111.5 (14)
N1—C2—C7	111.58 (14)	O3—Li1—H33	20.9 (10)
C2^ii^—C2—C7	129.53 (9)	N1—Li1—H33	123.2 (14)
O1—C7—O2	124.31 (16)	O1^i^—Li1—H33	78.6 (10)
O1—C7—C2	117.47 (16)	O1—Li1—H33	117.7 (10)

Symmetry codes: (i) −*x*+2, −*y*, *z*; (ii) *x*, *y*, −*z*.

### Hydrogen-bond geometry (Å, º)

**Table:**

*D*—H···*A*	*D*—H	H···*A*	*D*···*A*	*D*—H···*A*
O2—H1···O2^ii^	1.20 (1)	1.20 (1)	2.402 (3)	177 (8)
O3—H32···O3^iii^	0.87 (2)	2.00 (2)	2.839 (3)	163 (4)
O3—H31···O1^iv^	0.86 (2)	2.03 (2)	2.8825 (19)	177 (2)
O3—H33···O4^v^	0.86 (2)	2.10 (2)	2.9454 (17)	169 (6)

Symmetry codes: (ii) *x*, *y*, −*z*; (iii) −*x*+2, *y*, −*z*+1/2; (iv) *x*+1/2, −*y*+1/2, *z*; (v) −*x*+3/2, −*y*+1/2, −*z*+1/2.
